# Nicotinamide mitigates visceral leishmaniasis by regulating inflammatory response and enhancing lipid metabolism

**DOI:** 10.1186/s13071-024-06370-x

**Published:** 2024-07-06

**Authors:** Qi Zhou, Zhiwan Zheng, Shuangshuang Yin, Dengbinpei Duan, Xuechun Liao, Yuying Xiao, Jinlei He, Junchao Zhong, Zheng Zeng, Liang Su, Lu Luo, Chunxia Dong, Jianping Chen, Jiao Li

**Affiliations:** 1 Department of Pathogenic Biology, West China School of Basic Medical Sciences and Forensic Medicine, Sichuan University, Chengdu, Sichuan China; 2 Sichuan-Chongqing jointly-established Research Platform of Zoonosis, Chengdu, China; 3 Chong Qing Animal Disease Prevention and Control Center, Chongqing, China

**Keywords:** Visceral leishmaniasis, Nicotinamide, Immune response, Fatty acid degradation

## Abstract

**Background:**

Currently, treatment regimens for visceral leishmaniasis (VL) are limited because of the presence of numerous adverse effects. Nicotinamide, a readily available and cost-effective vitamin, has been widely acknowledged for its safety profile. Several studies have demonstrated the anti-leishmanial effects of nicotinamide in vitro. However, the potential role of nicotinamide in *Leishmania* infection in vivo remains elusive.

**Methods:**

In this study, we assessed the efficacy of nicotinamide as a therapeutic intervention for VL caused by *Leishmania infantum* in an experimental mouse model and investigated its underlying molecular mechanisms. The potential molecular mechanism was explored through cytokine analysis, examination of spleen lymphocyte subsets, liver RNA-seq analysis, and pathway validation.

**Results:**

Compared to the infection group, the group treated with nicotinamide demonstrated significant amelioration of hepatosplenomegaly and recovery from liver pathological damage. The NAM group exhibited parasite reduction rates of 79.7% in the liver and 86.7% in the spleen, respectively. Nicotinamide treatment significantly reduced the activation of excessive immune response in infected mice, thereby mitigating hepatosplenomegaly and injury. Furthermore, nicotinamide treatment enhanced fatty acid β-oxidation by upregulating key enzymes to maintain lipid homeostasis.

**Conclusions:**

Our findings provide initial evidence supporting the safety and therapeutic efficacy of nicotinamide in the treatment of *Leishmania* infection in BALB/c mice, suggesting its potential as a viable drug for VL.

**Graphical Abstract:**

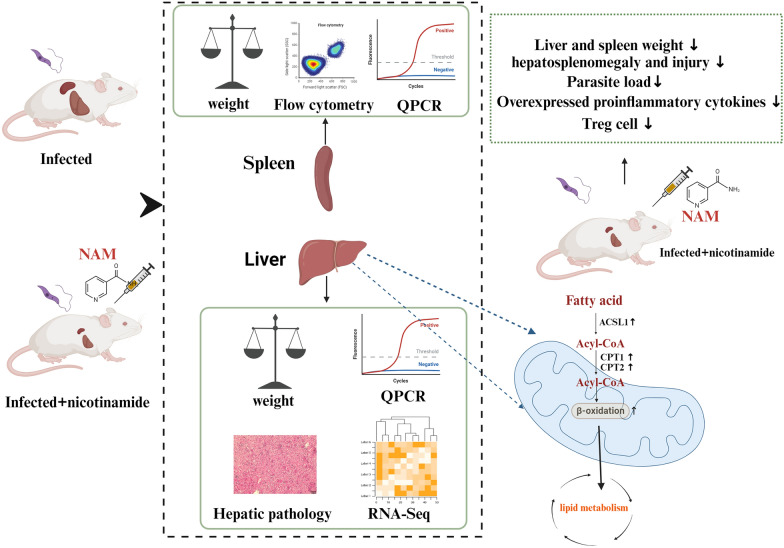

## Background

The World Health Organization (WHO) classifies leishmaniasis as a neglected tropical disease caused by the obligate intracellular parasite *Leishmania*, which is naturally transmitted by sandfly [1, 2]. The most severe and potentially fatal forms of this disease are known as visceral leishmaniasis (VL), primarily caused by *Leishmania infantum* and *L. donovani* [3]. The clinical presentation of VL typically includes recurrent episodes of fever, marked weight loss, hepatosplenomegaly, and severe anemia. The current situation indicates that VL remains a persistent global issue, with an estimated annual occurrence of 50,000–90,000 new cases [4]. This not only poses a significant threat to public health but also imposes a substantial economic burden on societies worldwide [5].

Currently, there is no universally accepted treatment guideline for leishmaniasis. Pentavalent antimony formulations have been recognized as the first-line therapeutic option since the 1920s. However, these drugs have been extensively documented to exhibit fatal hepato-, cardio-, and pancreatic toxicity [6]. The incidence of antimony resistance worldwide has witnessed a significant surge in recent times [7–9]. Alternative drugs for leishmaniasis, such as amphotericin B, miltefosine, paromomycin, pentamidine, and allopurinol, have demonstrated remarkable efficacy in treating the disease [10]. However, their utilization is limited because of toxic side effects, challenging administration methods, drug resistance issues, and high costs [11–13]. Therefore, it is imperative to explore an enhanced antileishmanial treatment that ensures improved safety profiles while remaining economically viable [14].

In addition to direct parasite elimination, new treatment strategies emphasize the mechanisms of host-parasite interactions and regulation of the host immune response. Nicotinamide (NAM), a water-soluble vitamin and essential component of vitamin B3, is an affordable and easily accessible option [15–17]. NAM is considered safe, even when administered at high dosages (6 g/day) in humans as it has no significant adverse effects [18, 19]. The utilization of NAM in high doses for various therapeutic applications has been extensive over the past few decades [17]. Extensive research has unequivocally demonstrated the remarkable antibacterial properties of NAM, showcasing its efficacy against a diverse range of pathogens including *Mycobacterium tuberculosis* [20], human immunodeficiency virus [21], *Candida albicans* [22], *Trypanosoma cruzi*, and *Plasmodium falciparum* [18, 19, 23]. The study conducted by Sereno et al. demonstrated that even at a concentration as low as 5 mM, NAM effectively inhibited the intracellular growth of *L. infantum* amastigotes [24]. The study conducted by Gazanion et al. further demonstrated the synergistic effect of NAM in combination with trivalent antimony, thereby enhancing its antileishmanial activity against *L. infantum* amastigote in vitro [25]. Additionally, Oliaee et al.’s research showed that the co-administration of NAM and Glucantime significantly reduces the proliferation of *Leishmania tropica* amastigotes within macrophages in vitro [26]. Despite several in vitro studies demonstrating the inhibitory potential of NAM against *leishmania* infection, there remains a dearth of in vivo investigations exploring its impact on *Leishmania* infection.

This study aimed to determine the immunomodulatory and protective effects of NAM against *L. infantum*-induced murine VL. Amphotericin B (AMB) was used as a positive control drug, and two control groups were included—a normal group and an infected-alone group. The efficacy of NAM was evaluated by assessing liver and spleen weight, parasite burdens, histopathological changes, spleen lymphocyte subset populations, and cytokine gene expression levels in each group. Additionally, transcriptome sequencing of liver tissues was conducted to investigate the intricate molecular mechanisms underlying the therapeutic efficacy of NAM.

## Methods

### Experimental animals

The female Balb/c mice (6–8 weeks old) weighing approximately 17–19 g were obtained from Dossy Experimental Animals Co., Ltd. (Chengdu, China) [27]. The animals were housed under standard conditions. A total of 21 mice were randomly divided into two groups: uninfected group (*n* = 12) and infected group (*n* = 9). The study protocol was approved by the Medical Ethics Committee of Sichuan University (approval number: KS2023517).

### In vivo toxicity assessment

To evaluate the hepatic and renal toxicity induced by NAM and AMB, the uninfected model mice (*n* = 9) were randomly divided into three groups: control group, control + NAM group (800 mg/kg body weight administered once daily intraperitoneally), and control + AMB group (1 mg/kg body weight administered every other day intraperitoneally). The treatment group received drugs for a duration of 14 days. Each group consisted of three biological replicates. Before euthanasia, blood samples were collected from experimental mice via retro-orbital puncture, followed by serum separation. Serum biochemistry parameters including alanine transaminase (ALT), aspartate aminotransferase (AST), urea nitrogen, and creatinine were measured according to the manufacturer's instructions (Shanghai Enzyme-linked Biotechnology Co., Ltd., China).

### *Leishmania infantum* infection and treatment

The mice in the infected group were intraperitoneally infected with 10^8^ *L. infantum* isolate (MHOM/CN/2016/SCHCZ, *L. HCZ*), which was obtained from the spleens and livers of infected hamsters. The animal groups were categorized as follows: group 1, control group; group 2, infected control group; group 3, infected and treated with NAM (800 mg/kg body weight, once a day intraperitoneally); group 4, infected and treated with AMB (1 mg/kg body weight, every alternative day intraperitoneally). Each group consisted of three biological replicates. On day 8 post-infection, the treatment groups received drugs for a duration of 14 days. After completion of treatment from the last doses (i.e. 21 days after infection), all animals were weighed and killed, and serum, liver, and spleen samples were collected for subsequent testing.

### Histopathological analysis

Some of the livers collected from killed mice were fixed in 10% paraformaldehyde, placed in alcohol to dehydrate embedded in paraffin wax, cut into sections, and then stained with H&E. Granulomas of pathological sections were observed under the optical microscope (Olympus, Japan) under 10 × and 40 × optical microscopy, respectively. The tissue sections and H&E staining were performed by Chengdu Aochuang Biological Company.

### Parasite load test

The parasite burdens in the spleen were assessed by calculating Leishman-Donovan units (LDU). The spleens were collected and weighed. Stamp smears of organs were prepared on clean, grease-free glass slides and stained with Giemsa to determine the number of amastigotes per thousand nucleated cells. The formula for LDU calculation is as follows: LDU = Number of amastigotes per 1000 nucleated cells × organ weight.

### Analysis of parasite burdens

The total RNA from tissue samples was extracted using the RNAeasy^™^ Animal RNA Isolation Kit (Beyotime, China) according to the manufacturer’s instructions. Subsequently, cDNA synthesis was performed using the BeyoRT^™^II First Strand cDNA Synthesis Kit (Beyotime, China). The concentration of resulting cDNA was determined using a NanoDrop One ultraviolet spectrophotometer (Thermo, USA) and adjusted to a concentration of 400 ng/μl. To evaluate parasite burdens in tissues further, RT-qPCR analysis was conducted to quantify the expression levels of *Leishmania* cysteine protease B genes (CPB). SYBR Green I dye (Beyotime, China) was employed for fluorescence detection. Primers were synthesized by Tsingke Biological Technology Co., Ltd. (Chengdu, China), as listed in Table 1. To more clearly illustrate the effects of NAM and AMB, the reduction rates were calculated in this study. Parasite reduction rate was calculated as the difference in parasite count between the infection and treatment groups, divided by the parasite count in the infection group.

**Table 1:** Part of the primers used in the study

Gene	Sequence (5′-3′)	Amplified gene size
CPB	AACGAAACGGTTATGGCTGC	128 bp
	CTTGTTGTACCCGACGAGCA	
IFN-γ	ATGAACGCTACACACTGCATC	182 bp
	CCATCCTTTTGCCAGTTCCTC	
TNF-α	CTTCTGTCTACTGAACTTCGGG	134 bp
	CAGGCTTGTCACTCGAATTTTG	
IL-6	ACAAGTCGGAGGCTTAATTACACAT	72 bp
	TTGCCATTGCACAACTCTTTTC	
IL-12	GCGGCATGTTCTGGATTTGACTC	132 bp
	CCACCACAGTTGCTGACTCATC	
IL-4	GGTCTCAACCCCCAGCTAGT	102 bp
	GCCGATGATCTCTCTCAAGTGAT	
IL-10	CGGGAAGACAATAACTGCACCC	138 bp
	CGGTTAGCAGTATGTTGTCCAGC	
IL-17	CAGACTACCTCAACCGTTCCAC	130 bp
	TCCAGCTTTCCCTCCGCATTGA	
Acaa1b	GGAGAATGTGGCTGAGCGGTTT	138 bp
	AGGACAGTGGTTGTCACAGGCA	
Acadm	AGGATGACGGAGCAGCCAATGA	120 bp
	GCCGTTGATAACATACTCGTCAC	
Acot1	AAGAAGCCGTGAACTACCTGCG	122 bp
	TGTGATGCCCTTCAGGAAGGAG	
ACSL1	ATCAGGCTGCTTATGGACGACC	130 bp
	CCAACAGCCATCGCTTCAAGGA	
CPT1α	GGCATAAACGCAGAGCATTCCTG	110 bp
	CAGTGTCCATCCTCTGAGTAGC	
CPT2	GATGGCTGAGTGCTCCAAATACC	99 bp
	GCTGCCAGATACCGTAGAGCAA	
CYP4A10	GCTACTCAAGGCTTTCCAGCAG	142 bp
	CCAGAACCATCTAGGAAAGGCAC	
CYP4A14	CAGCTACCAAGGCAGTGTTCAG	151 bp
	GGACAAACGTCCATCAGAGGAC	
Ehhadh	CAACTCCCTCAGGAGCATCTTG	144 bp
	GGTCTGACTCTACAGCAACCAC	
FABP1	AGGAGTGCGAACTGGAGACCAT	122 bp
	GTCTCCATTGAGTTCAGTCACGG	
GAPDH	TCTTGGGCTACACTGAGGAC	126 bp
	TCTTGGGCTACACTGAGGAC	

*CPB Leishmania* cysteine protease B, *IFN-γ* interferon gamma, *TNF-α* tumor necrosis factor alpha, *IL-6* interleukin-6, *IL-12* interleukin-12, *IL-4* interleukin-4, *IL-10* interleukin-10, *IL-17* interleukin-17, *Acaa1b* acetyl-coenzyme A acyltransferase 1B, *Acadm* acyl-coenzyme A dehydrogenase, medium chain, *Acot1* Acyl-CoA thioesterase 1, *ACSL1* Acyl-CoA synthetase long-chain family member 1, *CPT1α* carnitine palmitoyltransferase 1α, *CPT2* carnitine palmitoyltransferase 2, *CYP4A10* cytochrome P450, family 4, subfamily a, polypeptide 10, *CYP4A14* cytochrome P450, family 4, subfamily a, polypeptide 14, *Ehhadh* enoyl-coenzyme A, hydratase/3-hydroxyacyl coenzyme A, *FABP1* fatty acid binding protein 1, *GAPDH* glyceraldehyde-3-phosphate dehydrogenase

### Expression of fatty acid degradation related and cytokine genes

The expressions of seven cytokine genes (TNF-α, IFN-γ, IL-4, IL-6, IL-10, IL-12 and IL-17) and genes related to fatty acid degradation were quantified using RT-qPCR in all BALB/c mice. GAPDH was selected as the reference gene for normalization purposes. The relative expressions of these genes in the spleen and liver were determined using the 2 ^(−∆∆Ct)^ method by comparing them with a control group consisting of normal mice. Table 1 provides a list of all primers used in this study.

### Flow cytometry

To characterize spleen T lymphocyte subsets at the single-cell level, flow cytometry was performed for quantification of CD3^+^, CD3^+^CD4^+^, CD3^+^CD8^+^, and CD3^+^CD4^+^CD25^+^ T cells at the single-cell level in all groups of mice.

The spleens from each group were homogenized using a 70-μm cell filter and washed with normal saline and red cell lysate (Biosharp, China). A 10% fetal bovine serum solution was utilized to generate a cell suspension. Cells were then enumerated under a light microscope, with a concentration of 2 × 10^7^ cells/ml for each sample and subsequently stained with the following antibodies at 4 ℃ for 30 min in darkness: FITC Hamster Anti-Mouse CD3e (553061), APC Rat Anti-Mouse CD4 (553051), Ms CD8a PE (553032), BV421 Rat Anti-Mouse CD25 (564370), and 7-AAD (559925). T lymphocytes were detected using BD FACS Celesta, and data analysis was performed using FlowJo software (https://www.flowjo.com/).

### RNA-seq of liver tissue

Liver tissue transcriptome sequencing analysis was conducted by Shanghai Personalbio Technology Co., Ltd. RNA was extracted and purified, followed by library construction, and the Illumina sequencing platform was utilized for paired-end (PE) sequencing of these libraries using Next-Generation Sequencing (NGS) technology. Gene expression levels were calculated by comparing clean data obtained after filtering raw data with the reference genome (Mus musculus, GRCm39; dna.toplevel.fa). Based on the analysis results, further investigation was carried out on differentially expressed genes (DEGs) including differential expression analysis, functional enrichment analysis (KEGG and GSEA), and cluster analysis.

### Western blotting

The liver samples from each group were homogenized using a 70-μm cell filter and subsequently washed with normal saline and red cell lysate (Biosharp, China). The resulting precipitate was then lysed using RAIP lysate (Beyontime, China) along with a protein phosphatase inhibitor (MCE, USA). The extracted protein was quantified using the BCA kit (Beyotime, China). To denature the extracted proteins, an appropriate amount of loading buffer was added and the mixture was heated at 100 °C for 10 min. Subsequently, the proteins were separated via polyacrylamide gel electrophoresis and transferred onto PVDF membranes (Millore, USA). The PVDF membranes were incubated overnight at 4 ℃ with long-chain acyl-CoA synthetase 1 (ACSL1) antibody (Cell Signaling, USA, diluted to 1:1000) and β-actin antibody (Affinity, USA, diluted to 1:1000) at appropriate dilutions. Horseradish peroxidase-conjugated anti-rabbit immunoglobulin secondary antibodies as well as horseradish peroxidase-conjugated anti-mouse immunoglobulin secondary antibodies (Affinity, USA, diluted to 1:10000) were used as secondary antibodies for target protein detection.

### Statistical analysis

All statistical tests were performed using GraphPad Prism 7.04 (GraphPad Software Inc., USA). One-way or two-way ANOVA was used to determine the statistical significance of differences between multiple experimental groups. Data are expressed as mean ± SEMs. *P*-values were calculated and are indicated using asterisks as follows: *P < 0.05, **P < 0.01, ***P < 0.001.

## Results

### In vivo toxicity assessment of NAM

**Fig. 1:**
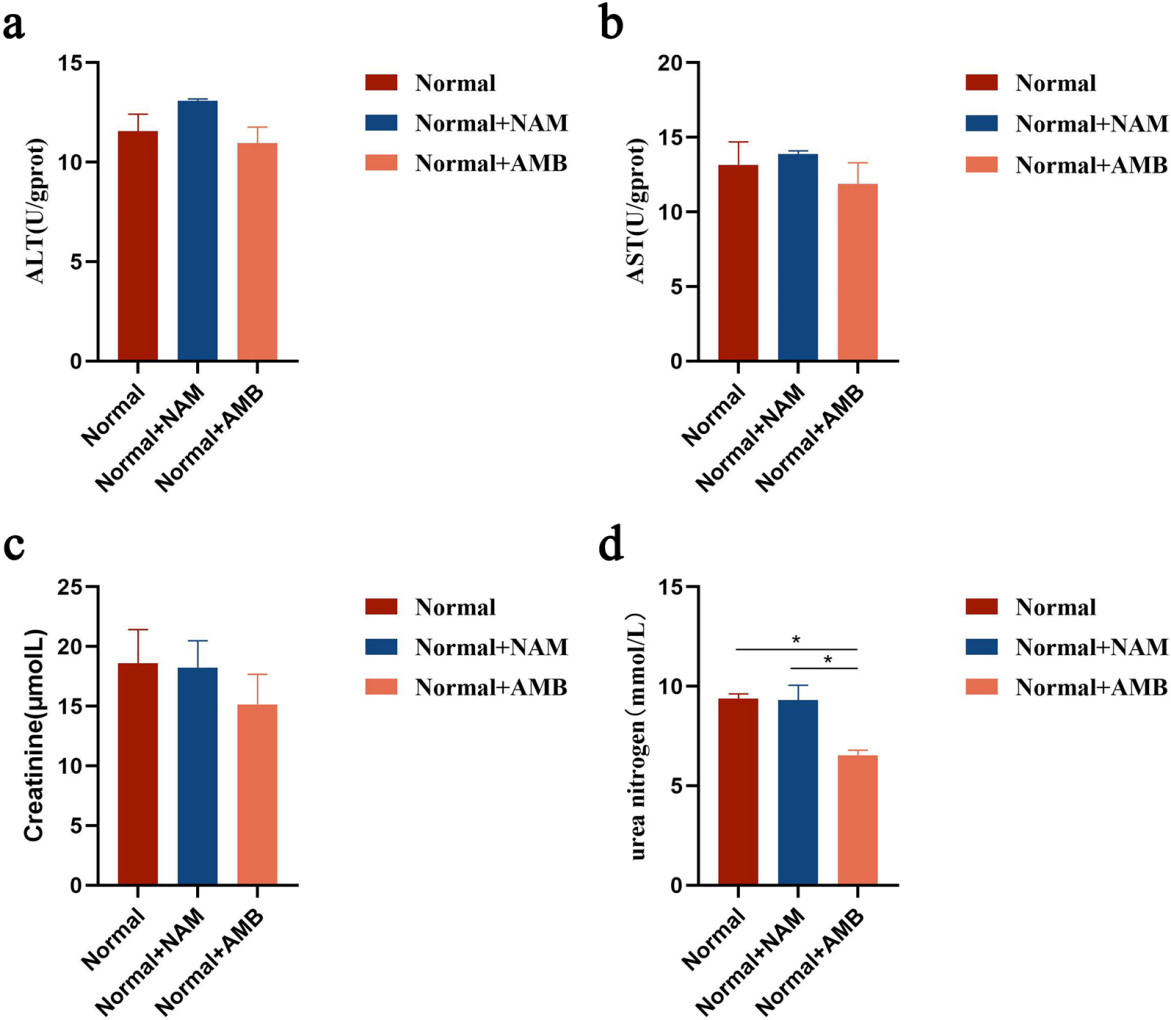
In vivo evaluation of the impact of NAM on liver and renal toxicity parameters in BALB/c mice. **a** Serum samples were analyzed for ALT. **b** Serum samples were analyzed for AST. **c** Serum samples were analyzed for creatinine. **d** Serum samples were analyzed for urea nitrogen. Control was normal mice without *Leishmania* infection. Results are expressed as mean ± SEM of *n* = 3 per group. *p < 0.05, **p < 0.01, ***p < 0.001, one-way ANOVA

The liver and kidneys are the primary organs targeted by drug-induced toxicity, and elevated levels of serum ALT, AST, creatinine, and urea nitrogen serve as indicators of hepatorenal toxicity. Our findings indicate that NAM treatment did not result in significant differences in ALT, AST, and creatinine levels among the three groups (Fig. 1a–c). However, the urea nitrogen level was higher in the NAM treatment group compared to the AMB group, although this difference was not statistically significant compared to the control group (Fig. 1d). These results demonstrate that the concentration of NAM used in this experiment is considered safe without any evidence of liver or kidney toxicity.

### NAM treatment effectively alleviates hepatosplenomegaly and injury in BALB/c mice

**Fig. 2:**
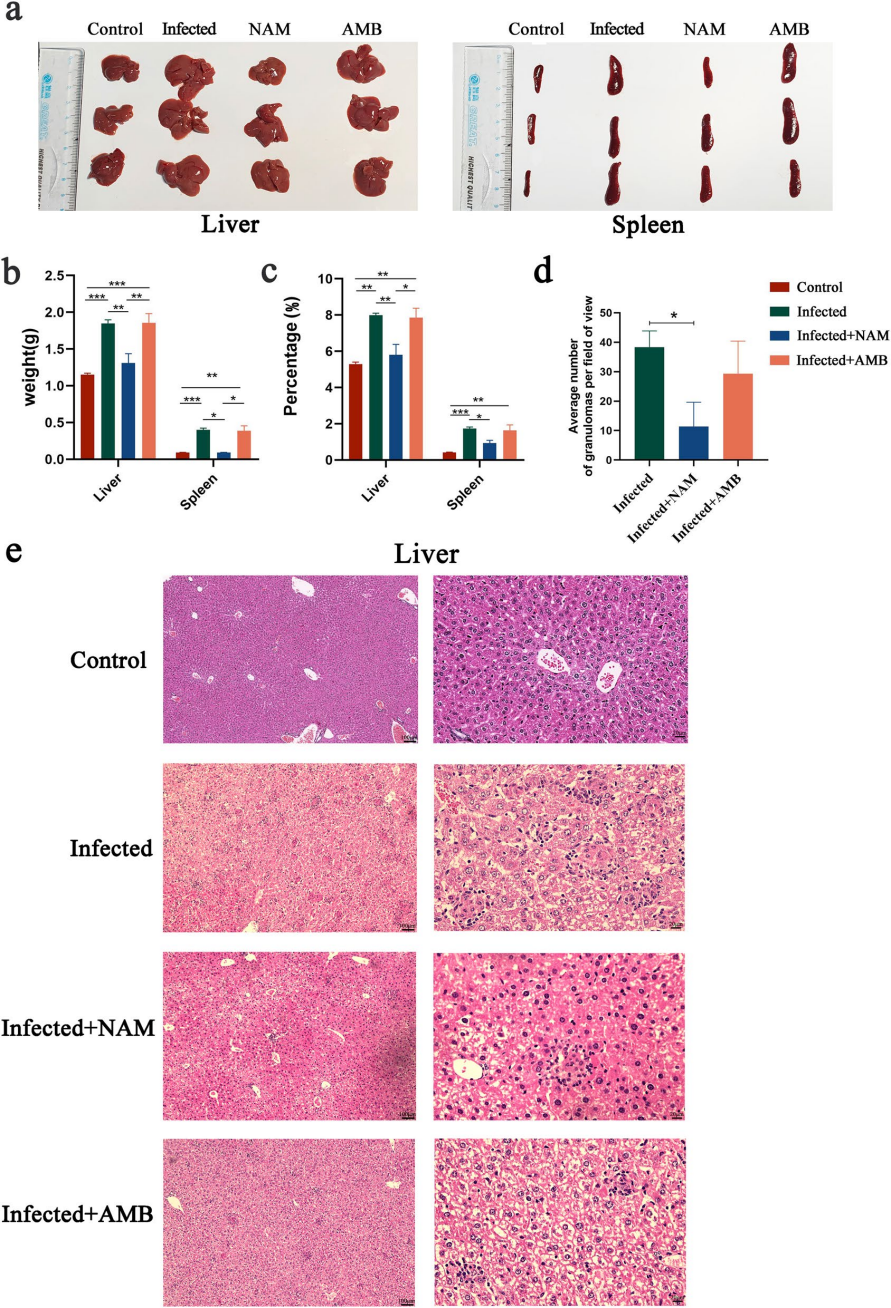
Morphological and hepatic histopathological alterations in the liver and spleen following NAM treatment in infected BALB/c mice. **a** Morphological changes of the liver and spleen were assessed in each group of mice 21 days after *Leishmania infantum* infection. **b** Liver and spleen weight changes in each group. **c** The ratio of liver and spleen weight to body weight of mice in each group. **d** Liver granuloma counts in infected, NAM, and AMB treatment group. **e** Hepatic pathology of mice in different groups. Images of hepatic granulomas from all groups at 100 × and 400 × the original magnification. Results are expressed as mean ± SEM of *n* = 3 per group. *p < 0.05, **p < 0.01, ***p < 0.001, one-way ANOVA

Swelling of the liver and spleen is a characteristic symptom of VL. Consequently, evaluating the degree of hepatosplenomegaly after treatment can be a valuable approach to assess the effectiveness of NAM. As shown in Fig. 2a, the administration of NAM significantly attenuated liver and spleen enlargement compared to the infected group, while AMB exhibited negligible impact. In line with the observed morphological alterations, a significant increase in liver and spleen weights was evident during *L*. *infantum* infection, followed by a decrease after NAM therapy (Fig. 2b). The viscera index was calculated to account for individual variations in weight. As illustrated in Fig. 2c, this observation is consistent with previous results. In addition to conducting morphological examinations, we also performed a histopathological analysis of the liver. As shown in Fig. 2d, e, granuloma formation and lymphocyte aggregation were observed in infected, NAM-treated, and AMB-treated groups. The infected group exhibited the highest number of granulomas and a high burden of amastigotes, while the NAM group primarily displayed mature granulomas and infiltration of inflammatory cells, indicating successful infection control. Furthermore, hepatocyte swelling was observed in all groups except for the control group, with the NAM group exhibiting the least amount of swelling. These findings suggest that NAM treatment effectively alleviates hepatosplenomegaly and injury in BALB/c mice.

### NAM exhibits in vivo antileishmanial activity against *L. infantum*

**Fig. 3:**
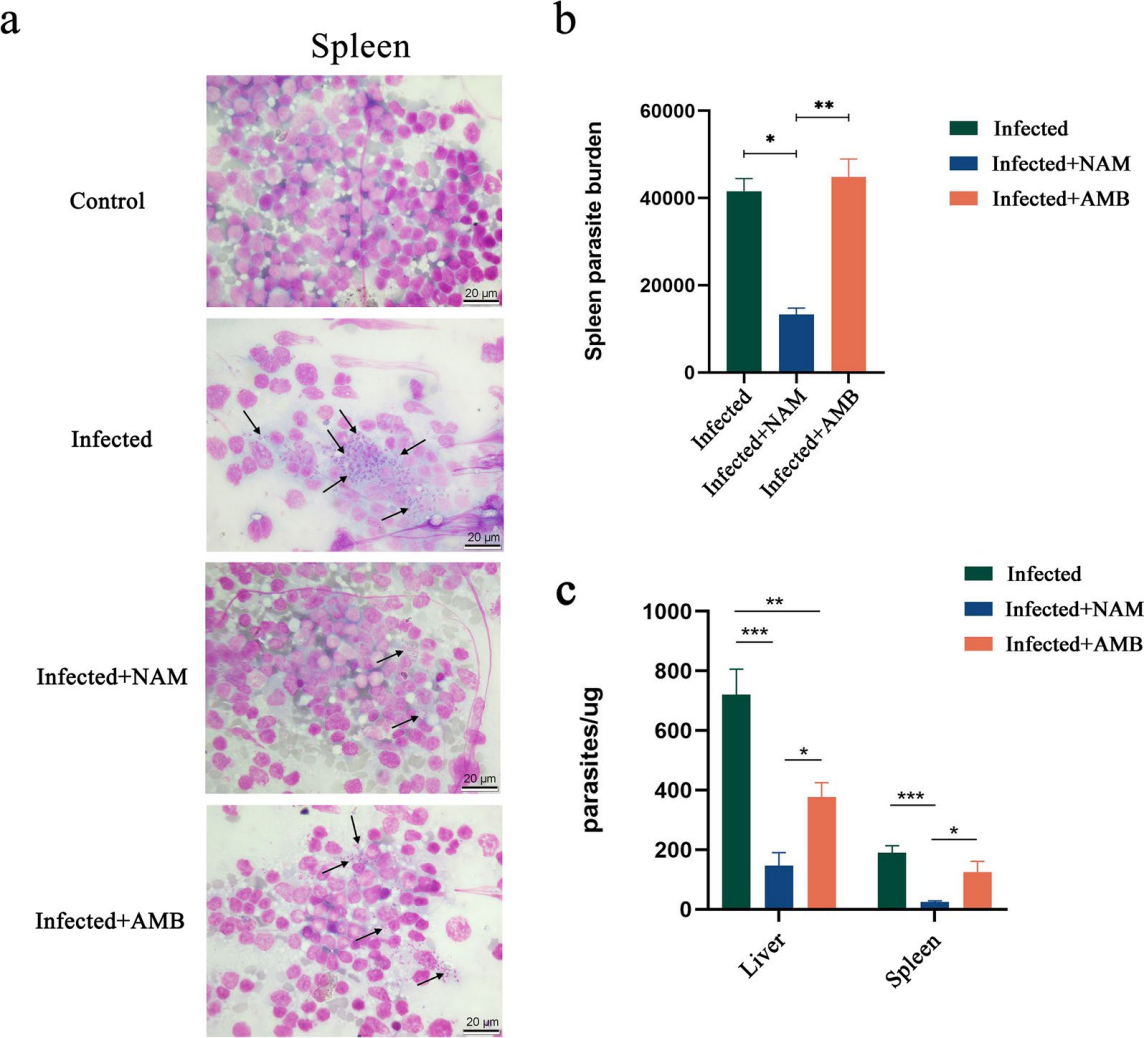
Parasite burdens of liver and spleen after NAM treatment in infected BALB/c mice. **a** Spleen imprints of BALB/c mice inoculated with *Leishmania infantum* after 21 days. **b** Spleen parasite burdens of infected, NAM-treated, and AMB-treated groups assessed by LDU. **c** The parasite burdens in the liver and spleen were assessed using real-time PCR for the infected, NAM-treated, and AMB-treated groups. Results are expressed as mean ± SEM of *n* = 3 per group. *p < 0.05, **p < 0.01, ***p < 0.001, one-way ANOVA

To assess the efficacy of NAM treatment against *L. infantum *in vivo, parasite burdens in spleen were evaluated by calculating the LDU. Compared to the infected control group, NAM treatment group showed a significant reduction compared to the AMB group (Fig. 3a, b). The efficacy of NAM against *L. infantum *in vivo was further confirmed by quantifying the parasite loads in the liver and spleen using qPCR. As anticipated, the NAM therapy group exhibited significantly lower levels of parasites in both organs compared to the group with a normal infection (Fig. 3c). To more clearly illustrate the effects of NAM and AMB, the parasite reduction rates were calculated. Compared to the control group, the reduction rates of parasites in the liver and spleen for the NAM group were 79.7 and 86.7%, respectively, while the AMB group had reduction rates of 47.6 and 33.9% in the same organs (Table 2). Collectively, these findings underscore the superior effectiveness of NAM over AMB.

**Table 2:** Amastigote reduction rate of liver and spleen

Drug	Liver (%)	Spleen (%)
NAM	79.7	86.7
AMB	47.6	33.9

*NAM* nicotinamide, *AMB* amphotericin B

### NAM effectively regulates the inflammatory response

**Fig. 4:**
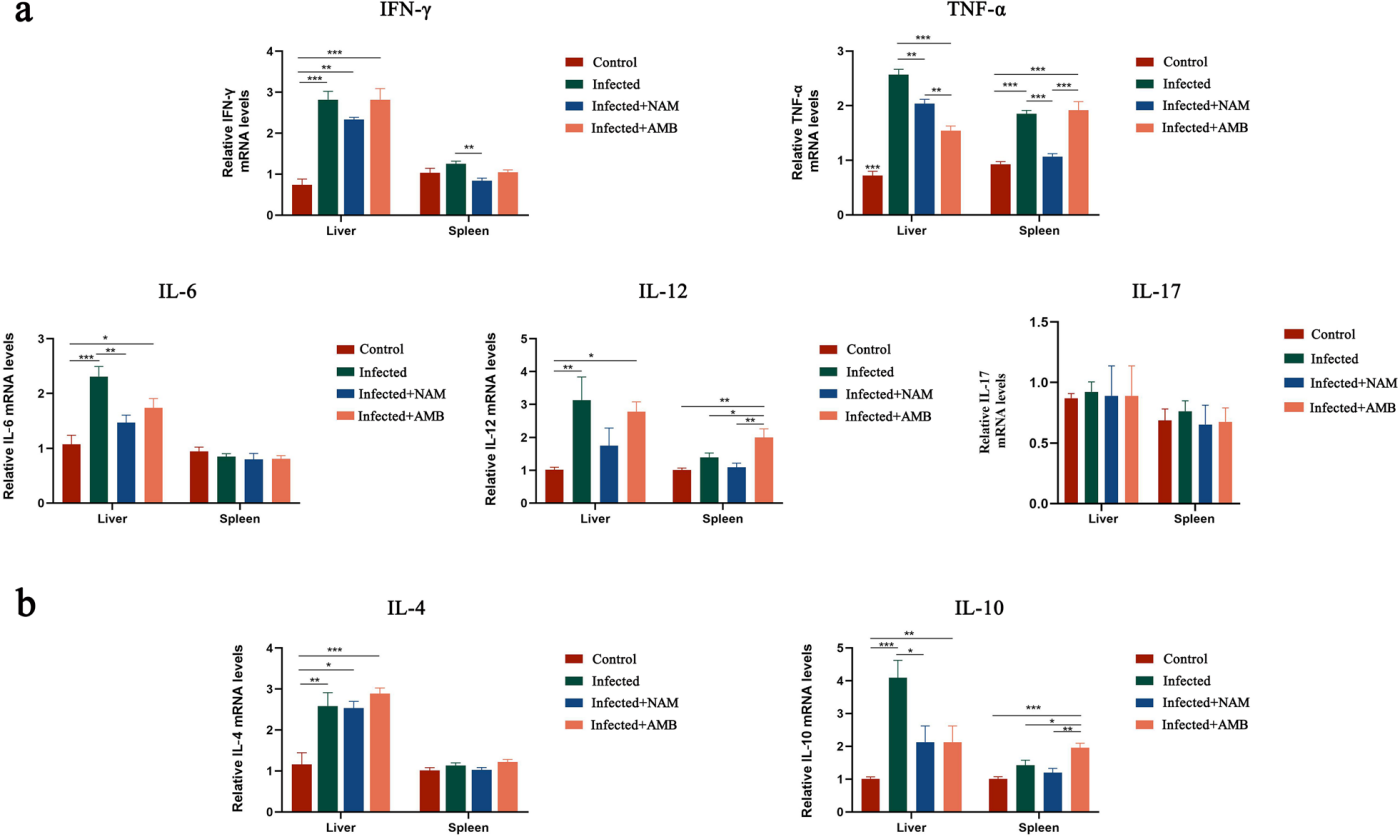
Regulation of cytokine secretion in the liver and spleen after NAM treatment. **a** Gene expression of IFN-γ, TNF-α, IL-6, IL-12, and IL-17. **b** Gene expression of IL-4 and IL-10. The relative expression levels of these genes were evaluated using real-time PCR, with normal mice employed as the control group. Results are expressed as mean ± SEM of *n* = 3 per group. *p < 0.05, **p < 0.01, ***p < 0.001, one-way ANOVA

To gain further insights into the potential mechanism by which NAM inhibits *Leishmania* infection, we compared mRNA expressions of seven cytokine genes (TNF-α, IFN-γ, IL-4, IL-6, IL-10, IL-12, and IL-17) in the liver and spleen of BALB/c mice. Overall, the liver exhibited more pronounced alterations in gene expression compared to the spleen (Fig. 4). In the spleen, mRNA levels of pro-inflammatory factors IFN-γ and TNF-α were significantly downregulated in the NAM group compared to the infected group. No statistically significant differences were observed in anti-inflammatory cytokine expression. In contrast, in the liver, hepatocytes from infected mice showed a significant increase in all six cytokines compared to those from control mice, except IL-17. This observation suggests that leishmaniasis triggers a robust immune response that may contribute to severe hepatomegaly and injury development. Subsequently, following NAM treatment, these cytokines were found to be downregulated to varying degrees with significant reductions observed for pro-inflammatory cytokines TNF-α and IL-6. These results suggest that NAM may alleviate excessive inflammatory responses by inhibiting cytokine secretion.

### T lymphocyte subsets in the spleen

**Fig. 5:**
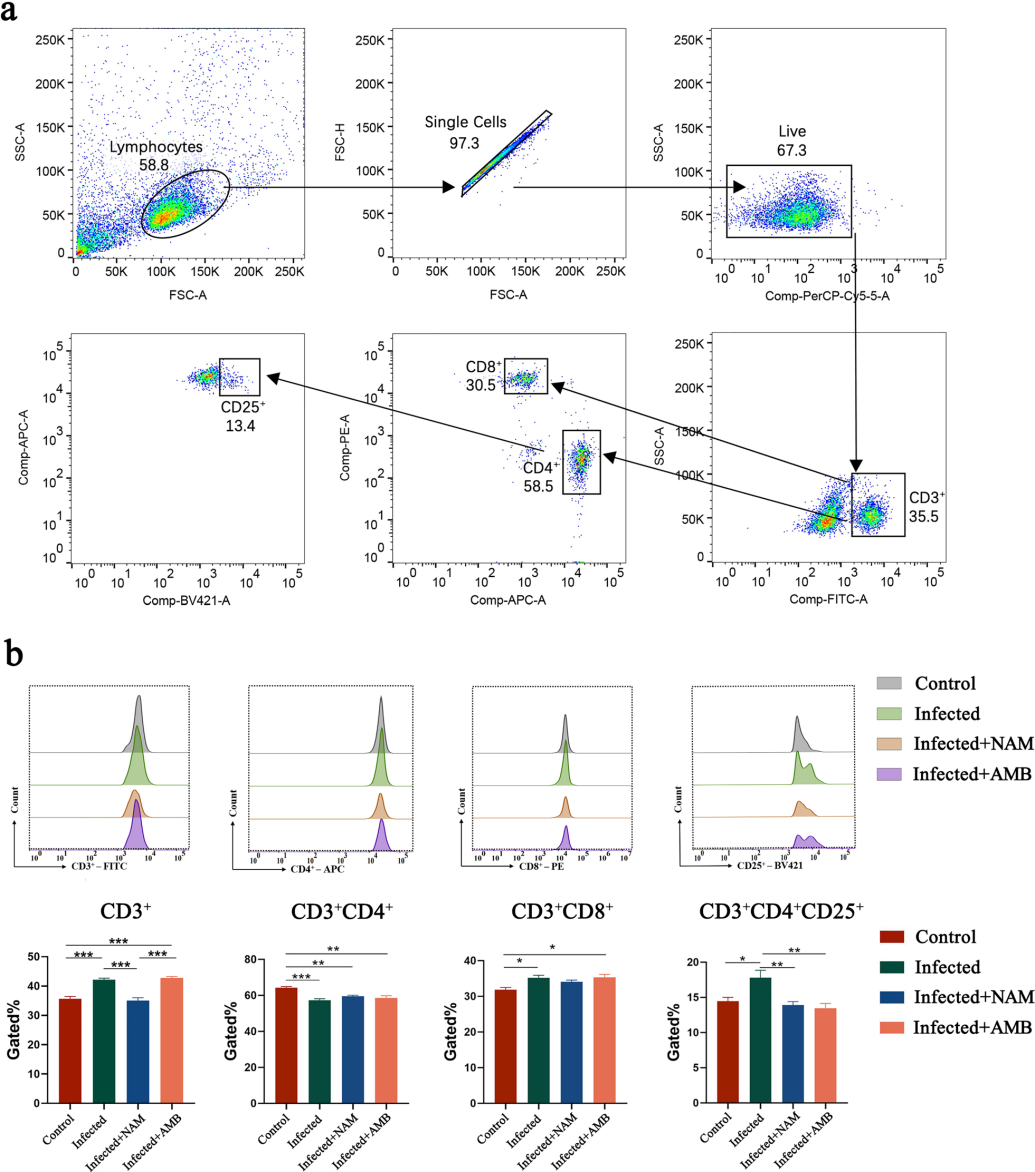
Flow cytometry results of spleen T lymphocytes after NAM treatment. **a** Gating strategy of spleen T lymphocyte flow cytometry. **b** Percent of CD3^+^, CD3 + CD4^+^, CD3^+^CD8^+^, and CD3^+^CD4^+^CD25^+^ T cells. Results are expressed as mean ± SEM of *n* = 3 per group. *p < 0.05, **p < 0.01, ***p < 0.001, one-way ANOVA

**Fig. 6:**
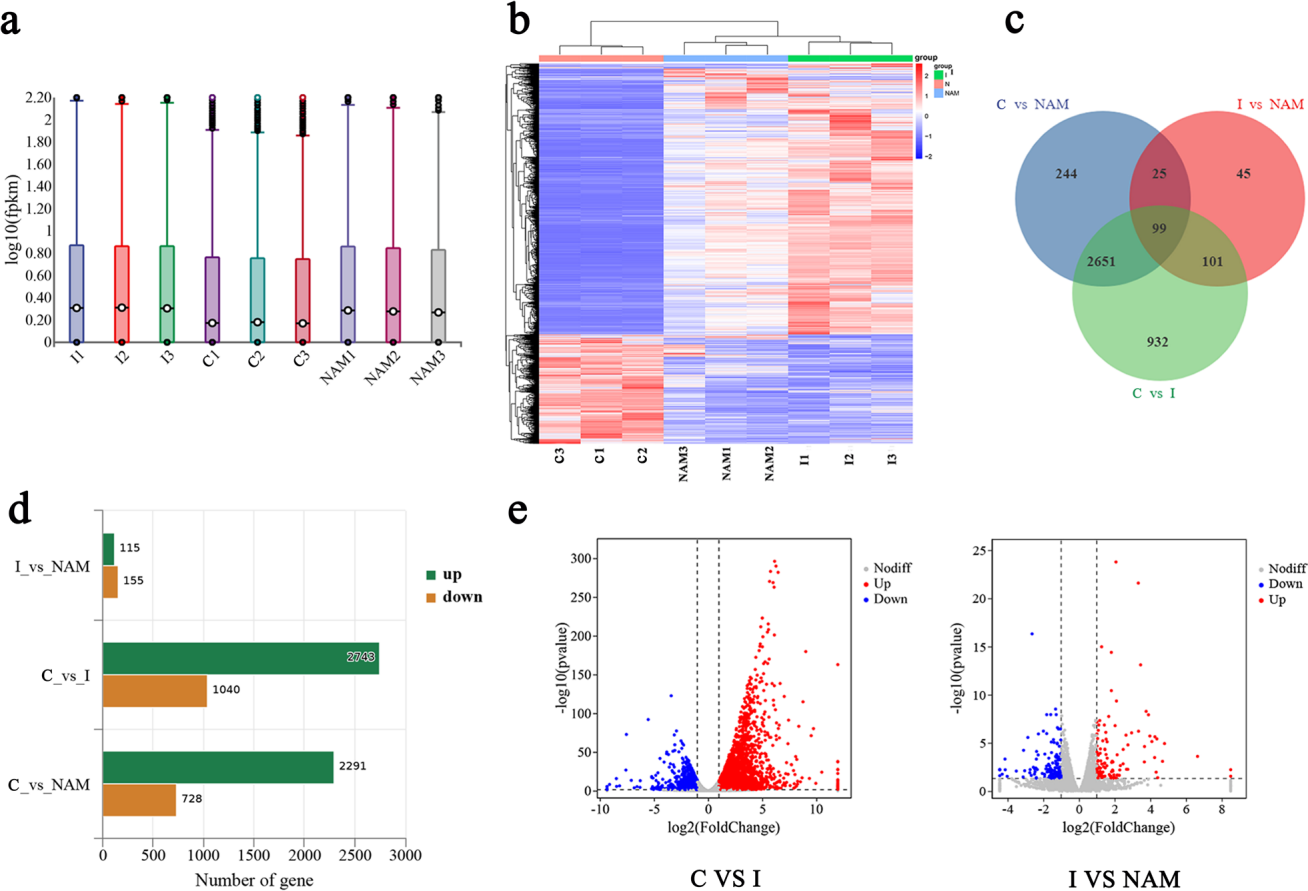
Differentially expression gene patterns and numbers in each group. **a** FPKM density violin plot for each sample. The horizontal line positioned at the center of the box represents the median, while the upper and lower boundaries of the box correspond to 75%, and the upper and lower limits are associated with 90%. **b** Cluster analysis of differentially expressed genes. The genes with high expression levels are depicted in the color red, while those with low expression levels are represented by the color blue. **c** Venn diagram shows the common differentially expressed genes in different groups. **d** The bar graph shows the number of up- and downregulations of differentially expressed genes between different groups. **e** Volcano plot of DEGs between different groups. Red: upregulated, blue: downregulated, gray: no difference

The spleen, a vital peripheral immune organ in the human body, plays a crucial role in the proliferation of various immune cell populations. Therefore, flow cytometry was employed in this study to characterize T lymphocyte subsets within the spleen. Figure 5a illustrates the gating strategy used for CD3^+^, CD3^+^CD4^+^, CD3^+^CD8^+^, and CD3^+^CD4^+^CD25^+^ T cell flow cytometry analysis. The distribution of different T lymphocyte populations is presented in Fig. 6b. Statistical analysis revealed a significant decrease in total lymphocytes (CD3^+^) within the NAM group compared to the infection group. However, no significant differences were observed between the NAM group and infected group regarding CD3^+^CD4^+^ and CD3^+^CD8^+^ subsets. Regulatory T cells, known as Tregs (CD3^+^CD4^+^CD25^+^), exhibited a significant upregulation following infection but showed a notable downregulation after treatment with NAM or AMB.

### Comprehensive analysis of gene expression patterns using RNA sequencing technology

As the largest metabolic organ, the liver plays an important role in body metabolism [28]. VL can induce hepatomegaly and liver damage, leading to a series of metabolic changes such as hypocholesterolemia, lipoprotein alteration, and hypertriglyceridemia [29]. To elucidate the intricate molecular mechanisms underlying liver dysfunction caused by *Leishmania* infection, RNA-seq was employed to investigate changes in hepatic gene expression among control mice, infected mice, and mice treated with NAM. The quality of the raw sequencing data for the three groups of samples was assessed, and Q20 and Q30 values exceeding 98 and 97%, respectively, were obtained. These results suggest that base identification accuracy was at least 99 and 99.9%. The FPKM density violin plot revealed a consistent gene distribution pattern in each group, with most genes exhibiting moderate expression levels with fewer genes displaying high or low expression levels. These results indicate that RNA-seq data are of high quality and suitable for the subsequent analysis (Fig. 6a). Clustering analysis of DEGs demonstrated effective grouping of biological replicates within each group, revealing significant differences in gene expression among different groups (Fig. 6b). The Venn diagram illustrates unique and shared DEGs between various groups (Fig. 6c). Figure 6d demonstrates the presence of DEGs between the infection group and NAM group, with 115 upregulated and 155 downregulated genes. The volcano plot shows significantly up- or downregulated DEGs detected between the various groups; red indicates upregulation while blue indicates downregulation (Fig. 6e).

### Function enrichment analysis: KEGG and GSEA

**Fig. 7:**
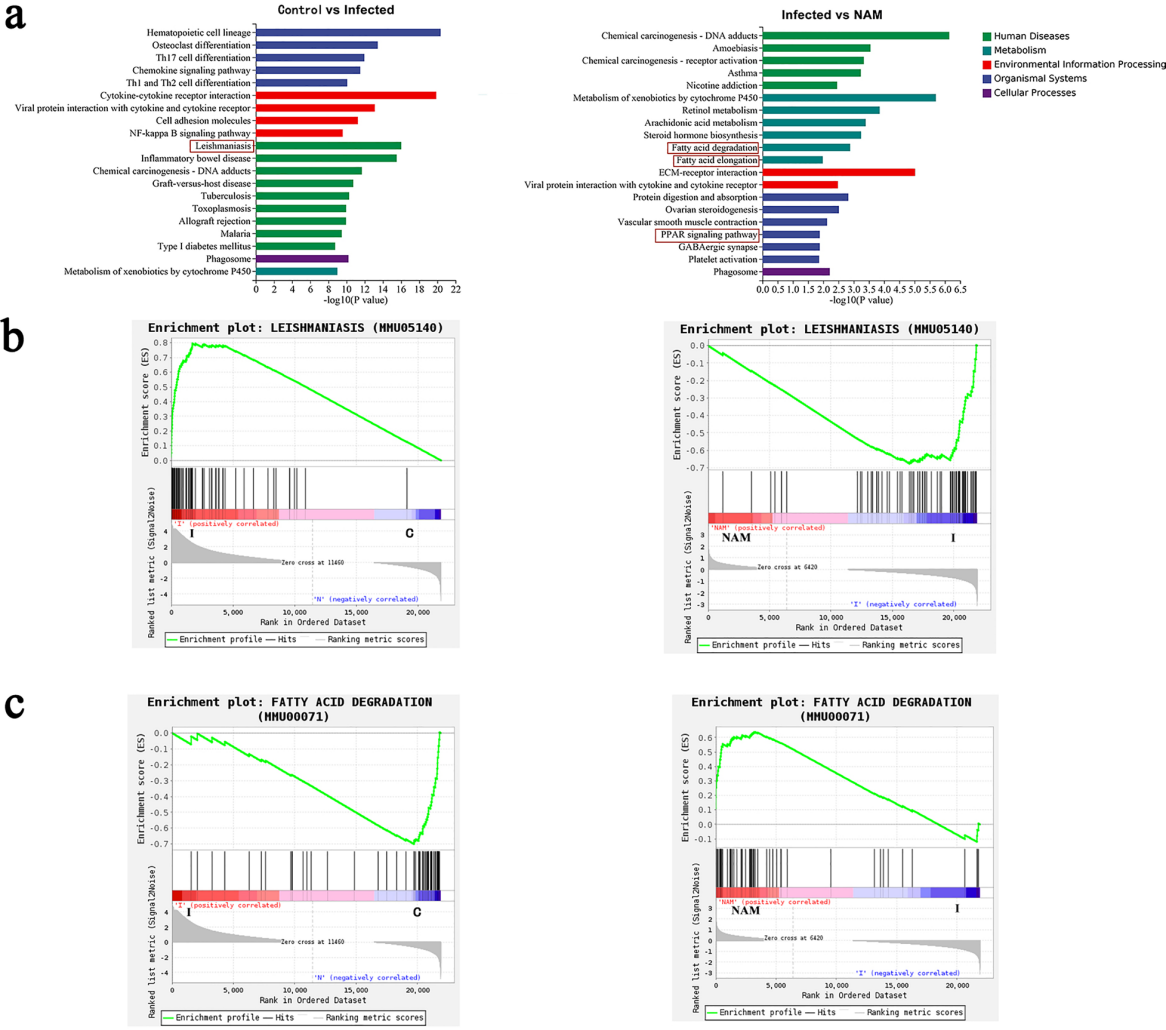
KEGG and GSEA enrichment analysis on the DEGs between control and infected groups and infected and NAM group. **a** KEGG classifications of the DEGs. The horizontal axis is the pathway, and the vertical axis is -log^10^ of pathway enrichment (*p*-value). **b**, **c** GSEA enrichment analysis results of leishmaniasis and fatty acid degradation

To gain a comprehensive understanding of the potential functions of DEGs and elucidate the underlying mechanism of NAM in resisting *Leishmania* infection, we conducted KEGG functional enrichment analyses based on the identified DEGs. Figure 7a provides an overview of the top 20 enriched KEGG pathways in pairwise comparisons between various groups. The analysis revealed that DEGs were predominantly associated with immune-related pathways, including chemokine signaling, Th1 and Th2 cell differentiation, cytokine-cytokine receptor interaction, NF-κB signaling, and leishmaniasis in the infected group compared to the control group. Notably, after treatment with NAM, significant changes in DEGs were primarily observed in pathways related to lipid metabolism including fatty acid degradation, fatty acid elongation, and the PPAR signaling pathway (Fig. 7a). To further identify differentially regulated gene sets across groups, Gene Set Enrichment Analysis (GSEA) was performed. The results demonstrated downregulation of the leishmaniasis gene set, while upregulation was observed for the fatty acid degradation gene set following NAM treatment (Fig. 7b, c). Based on these findings from KEGG functional enrichment analyses and GSEA results, we propose that NAM may potentially act against the infection of *Leishmania* by modulating host immune response and fatty acid metabolism.

### Validation of the RNA-seq results by qPCR and WB

**Fig. 8:**
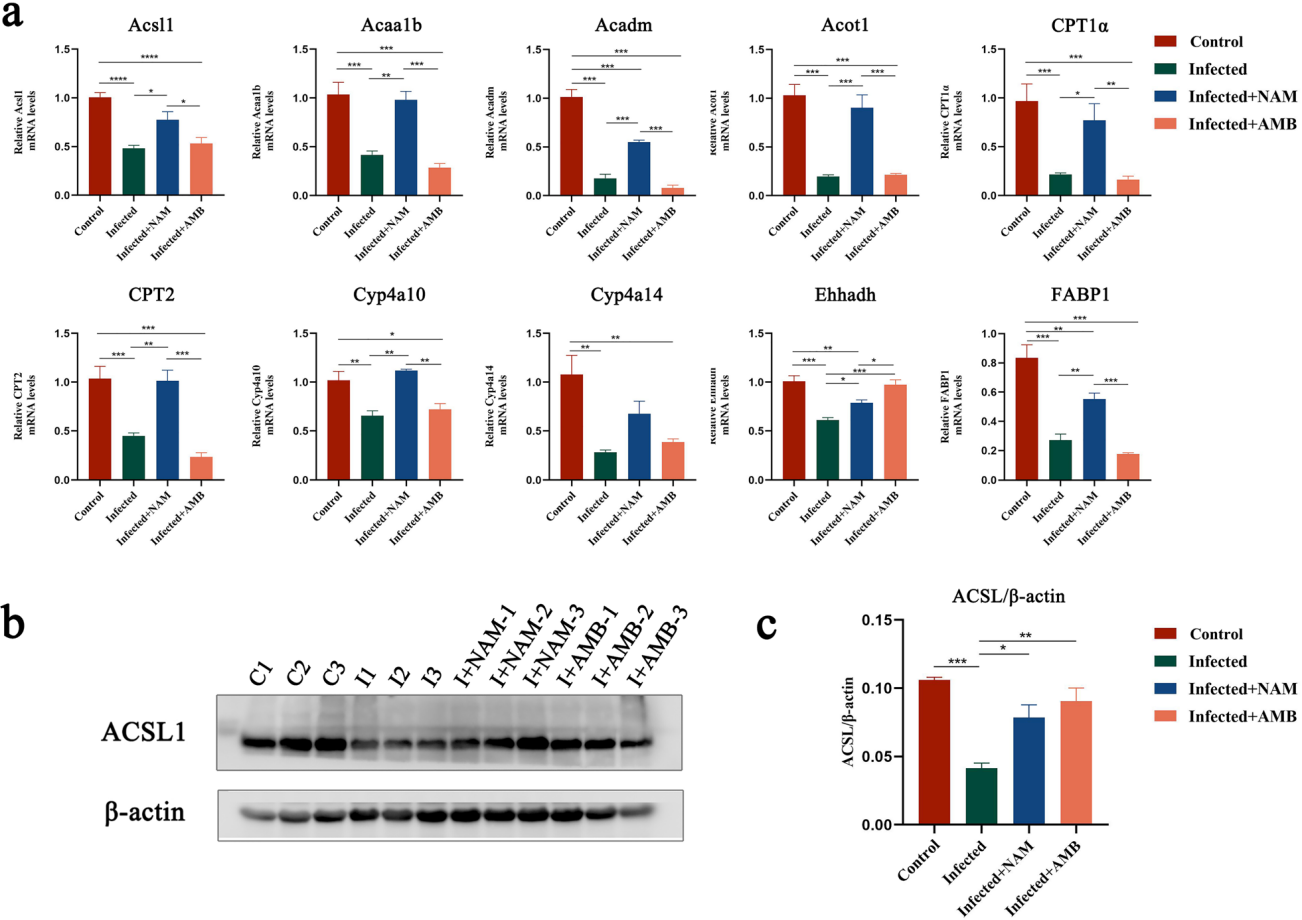
Quantification of hepatic expression levels of enzymes involved in fatty acid degradation. **a** Gene expression of fatty acid degradation-related genes was measured by RT-qPCR. **b** Hepatic ACSL1 expression levels of mice in each group after NAM treatment. **c** Relative fold change in ACSL1 expression, normalized by an endogenous control, β-actin. Results are expressed as mean ± SEM of *n* = 3 per group. *p < 0.05, **p < 0.01, ***p < 0.001, one-way ANOVA

The KEGG functional enrichment analyses revealed that the changes in DEGs were primarily enriched in pathways associated with lipid metabolism following NAM treatment. Fatty acid oxidation, a pivotal process in lipid metabolism and lipid homeostasis, plays a crucial role. In this study, qPCR was employed to validate the expression levels of 10 genes involved in fatty acid oxidation, namely Acaa1b, Ehhadh, Cyp4a14, Cyp4a10, CPT2, FABP1, CPT1α, ACSL1, Acot1, and Acadm (Fig. 8a). Consistent with the transcriptome results obtained earlier in our investigation, our findings revealed a significant downregulation of these ten genes following infection and an upregulation of their expression after treatment with NAM. Notably, ACSL1 plays a pivotal role as the key enzyme initiating fatty acid oxidation. Therefore, WB analysis was performed to further assess its protein expression level, which demonstrated a decrease following infection but subsequently increased upon NAM treatment (Fig. 8b, c).

## Discussion

NAM, a water-soluble vitamin, exhibits antibacterial activity against a diverse range of pathogens. NAM has been widely regarded as safe, with a low occurrence of side effects and toxicity [15]. In our study, drug toxicity of NAM was performed using a normal mouse model. Analysis of four biochemical indicators, namely ALT, AST, creatinine, and urea nitrogen, as markers of hepatorenal toxicity, revealed no significant difference between the NAM group and control group, further confirming the safety profile of NAM. On the other hand, we established a mouse model infected with *L. infantum* and utilized AMB as a positive control to investigate whether NAM exhibits inhibitory effects on *leishmania* infection in vivo. Our findings demonstrated that compared to AMB treatment, NAM exhibited more pronounced efficacy in alleviating hepatosplenomegaly and injury in BALB/c mice. Furthermore, results from LDU and qPCR assays indicated that NAM effectively reduced parasite loads. These results underscore the therapeutic potential of NAM for treating VL caused by *L. infantum*.

The infection of *Leishmania* typically elicits a robust inflammatory response accompanied by varying degrees of tissue damage [30, 31]. The results of this study revealed a significant upregulation of pro-inflammatory cytokines, including TNF-α, IFN-γ, and IL-6, in the *Leishmania*-infected group. This was accompanied by a notable increase in the number of liver granulomas. The robust inflammatory response observed contributed to the pronounced enlargement of the liver and spleen. In contrast, the NAM treatment group exhibited a marked reduction in cytokine secretion and granuloma formation, resulting in mitigated hepatosplenomegaly and diminished tissue injury. It is now widely acknowledged that maintaining an appropriate balance between pro- and anti-inflammatory cytokines is crucial for achieving a cure and preventing immune-mediated pathological damage or disease reactivation. Currently, numerous studies have demonstrated the ability of NAM to inhibit inflammation through diverse mechanisms [32, 33]. Consistent with our research, Zheng et al. found that NAM alleviated renal interstitial fibrosis by suppressing T cell and macrophage infiltration as well as inhibiting proinflammatory cytokines such as TNF-α and IL-1β [34]. The study conducted by González et al. demonstrated that NAM possesses the capability to mitigate inflammation and oxidative stress in metabolic syndrome-induced rats through modulation of the cholinergic system [35]. IL-6, a pleiotropic cytokine, plays a pivotal role in various processes encompassing pathogen infection and immune system activation [36]. In leishmaniasis, IL-6 has been detected in the serum of patients with both visceral and cutaneous forms of the disease and exhibited significant downregulation following treatment [37]. TNF-α, primarily secreted by macrophages and monocytes, plays a significant role in the initiation and progression of inflammatory processes [38, 39]. Sukhumavasi et al. demonstrated an association between excessive production of TNF-α and structural damage as well as immune dysfunction in chronic inflammation caused by *Leishmania* infection [40]. Zhang et al.’s research revealed that NAM effectively reduces acute lung injury by significantly suppressing the gene expression of IL-6, IL-1β, and TNF-α [41]. Based on the aforementioned findings, we postulate that in this study the reduction of parasite load in the NAM treatment group may be attributed to the modulation of pro-inflammatory cytokine expression, thereby mitigating hepatosplenomegaly and injury resulting from excessive inflammation.

The flow cytometry results from this study demonstrate a significant decrease in CD3^+^ T cells following NAM treatment compared to the infected group. Conversely, no significant differences were observed in CD4^+^ and CD8^+^ T cells. It is hypothesized that the reduction in CD3^+^ T cells may be associated with the marked decrease in expression of CD25^+^ T cells. Regulatory T cells, known as Tregs (CD3^+^CD4^+^CD25^+^), contribute to persistent infection by facilitating parasite evasion [42, 43]. Previous studies have indicated that increased activation of Tregs in cutaneous leishmaniasis patients impairs parasite elimination, leading to the establishment of chronic infection [44]. The flow cytometry results obtained in this study reveal a significant increase in the percentage of Tregs following infection, which was significantly reduced after treatment with NAM. Eufrásio et al. found that reducing the number of splenic Treg cells was associated with decreased parasite burdens and splenomegaly in BALB/c mice infected with *L. infantum* [45]. The precise mechanism by which NAM exhibits antileishmanial activity remains elusive. However, there is a possibility that NAM functions as an indirect antibacterial agent through regulating Treg cells. Further research is needed to gain a better understanding of these aspects.

Lipids are essential components involved in infection and inflammation, playing a crucial role in the interactions between hosts and pathogens [46]. Zhang points out that intracellular amastigotes primarily acquire lipids from the host to facilitate the membrane formation and serve as an energy source [47]. *Leishmania* manipulates host metabolic flux (including lipid metabolism) as a strategy to evade host immune responses, enabling long-term parasite survival [48]. Previous research has consistently demonstrated that *Leishmania* infection highly perturbed host lipid metabolic pathways, resulting in liver dysfuntion [47, 49, 50]. In this study, transcriptome analysis was conducted on liver samples from three groups: the control group, infected group, and NAM group. Our findings revealed that *L. infantum* infection downregulates the fatty acid degradation pathway. However, interestingly, this pathway was upregulated after NAM treatment. Fatty acid oxidation, a crucial process in fatty acid degradation, plays a vital role in maintaining fatty acid metabolism and lipid homeostasis [51]. A diverse array of enzymes participates in the intricate process of fatty acid oxidation. By integrating RNA sequencing and qPCR results, we have demonstrated that treatment with NAM upregulates pivotal enzymes involved in fatty acid oxidation, CPT1 and CPT2 serve as the rate-limiting enzymes in fatty acid β-oxidation, and augmenting their activity and levels can enhance hepatic function and ameliorate liver injury [52, 53]. In mice infected with *Schistosoma japonicum*, praziquantel has been demonstrated to effectively alleviate liver injury by upregulating the expression of Cyp4a14 [54]. Murine deficiency of EHHADH disrupts hepatic cholesterol homeostasis [55]. Similarly, downregulation of ACADM expression impedes fatty acid β oxidation, thereby facilitating hepatocellular invasion and promoting liver cancer progression [56]. ACSL1 is responsible for catalyzing free fatty acids (FFAs) into fatty acyl-CoA (FA-CoA), playing a critical role in lipid homeostasis and serving as the key enzyme initiating fatty acid oxidation [50]. Research has indicated that a deficiency in ACSL can exacerbate liver damage, resulting in outcomes such as increased liver weight, elevated AST/ALT levels, and heightened inflammatory responses; this aligns with the liver phenotype observed during VL infection [57]. In this study, WB analysis revealed a decrease in ACSL1 protein levels following infection but an increase after NAM treatment. These findings suggest that ACSL1 may contribute to liver injury associated with VL. Previous studies on *Trypanosoma cruzi*, which belongs to the same family as *Leishmania*, have indicated that carnitine (coenzyme for fatty acid oxidation) can regulate acylcarnitine metabolism disorders, making it a promising therapeutic approach for the acute phase of Chagas disease [58]. In conclusion, our findings suggest that NAM has the potential to act against *Leishmania* infection by facilitating fatty acid oxidation and enhancing lipid metabolism.

AMB, a macrolide polyene antifungal drug, serves as a second-line treatment for VL by inhibiting ergosterol synthesis and disrupting the parasite’s cell membrane [59, 60]. The existence of AMB-resistant strains of *Leishmania* has been demonstrated in several reports [61–63]. In contrast to NAM treatment, AMB failed to mitigate the excessive production of cytokines such as TNF-α and IL-12, leading to an inability to alleviate hepatosplenomegaly and inflammatory injury in BALB/c mice. Furthermore, LDU and qPCR results indicated that AMB only reduced parasite load in the liver compared to the infected group. We propose several potential reasons for this phenomenon. First, variations in susceptibility and drug resistance among *Leishmania* isolates are commonly observed because of differences in species and geographical location [61]. The strain of *Leishmania* utilized in our study, *L. HCZ*, is a novel isolate obtained from the bone marrow of a kala-azar patient by our research team. Previous reports indicated that this strain exhibits high virulence and notable drug resistance [64, 65], potentially contributing to its diminished susceptibility to AMB. Second, the significant side effects and toxicity associated with AMB limit its clinical utility. Consequently, efforts to enhance its efficacy and mitigate its toxicity have primarily focused on liposomal formulations of AMB [66]. Research suggests that liposomal formulations are more readily absorbed and demonstrate improved efficacy compared to traditional AMB formulations [60]. Due to limitations in its mode of administration (intravenous injection), the non-liposomal form of AMB was used in this study. This could result in suboptimal absorption, leading to less-than-ideal efficacy. Third, organ-specific and other pathophysiological factors can influence the specific distribution and elimination of drugs across various organs, resulting in varying degrees of parasite reduction [67].

## Conclusions

Consequently, our findings provide initial evidence of the safety and therapeutic efficacy of NAM in treating *L. infantum* infection in BALB/c mice, thereby presenting a promising avenue for the development of innovative therapeutic strategies against VL.

In this study, our focus was solely on evaluating the therapeutic effect of NAM against *L. infantum* infection model. Future research should aim to validate its efficacy against other strains of leishmania. Furthermore, subsequent studies can explore the potential synergistic effects of combining NAM with other drugs.

## Data Availability

Data will be made available on request.

## Abbreviations

VL: Visceral leishmaniasis
NAM: Nicotinamide
AMB: Amphotericin B
ALT: Alanine transaminase
AST: Aspartate aminotransferase
LDU: Leishman-Donovan units
CPB: Cysteine protease B genes
NGS: Next-generation sequencing
DEGs: Differentially expressed genes
